# Supplementary material for: Magic-BLAST, an accurate RNA-seq aligner for long and short reads
Source: BMC Bioinformatics. 2019 Jul 25;20:405. doi: 10.1186/s12859-019-2996-x (PMC6659269; doi:10.1186/s12859-019-2996-x)
Supplement: Supplementary file 1 — The supplement consists of a document with seven sections, successively 1- Bioinformatics (aligners and analysis programs), 2- datasets and length histograms, 3- introns discovered per coverage (ROC curves), 4- introns precision and recall, 5- iRefSeq results, 6- alignment mapping precision and recall measured by comparison to the benchmark, 7- alignment statistics and quality control. Additionally, a number of excel spread sheets with underlying data have been provided. All comments are welcome. (ZIP 3341 kb) [file 12859_2019_2996_MOESM1_ESM.zip › Magic-BLAST_Supplementary_material.docx]

# Supplementary material for: **Magic-BLAST, an accurate DNA and RNA-seq aligner for long and short reads**

Grzegorz M Boratyn, Jean Thierry-Mieg, Danielle Thierry-Mieg, Ben Busby and Tom Madden

Please contact [mieg@ncbi.nlm.nih.gov](mailto:mieg@ncbi.nlm.nih.gov) for any question about the supplementary material or the data analysis.

Contents

[Supplementary material for: **Magic-BLAST, an accurate DNA and RNA-seq aligner for long and short reads** 1](#_Toc526994667)

[1- Bioinformatics 2](#_Toc526994668)

[1.1 Magic-BLAST command line parameters 2](#_Toc526994669)

[1.2 Aligners tested, parameters and performance 2](#_Toc526994670)

[1.3 Other Programs 5](#_Toc526994671)

[1.4 Description of the analysis. 6](#_Toc526994672)

[1.5: How to reproduce this analysis. 6](#_Toc526994673)

[2-Datasets 7](#_Toc526994674)

[2.1 Construction of the iRefSeq 7](#_Toc526994675)

[2.2 Description of additional datasets tested 7](#_Toc526994676)

[2.3 Histograms of length to be aligned and effectively aligned by each program 8](#_Toc526994677)

[3-Introns discovered, per coverage (ROC curves) 14](#_Toc526994678)

[3.1 Supporting data for the ROC curves: 14](#_Toc526994679)

[3.2 Graphical representations for additional runs and zoomed views: 16](#_Toc526994680)

[4- Intron precision and recall 21](#_Toc526994681)

[4.1 All experimental datasets and iRefSeq 21](#_Toc526994682)

[4.2 The Human Baruzzo benchmark (100+100) and the human T1 run truncated at 50+50 23](#_Toc526994683)

[4.3 The malaria Baruzzo benchmark and the 1/100 subsampled malaria run 26](#_Toc526994684)

[5- Results on the iRefSeq dataset 28](#_Toc526994685)

[6- Mapping accuracy by comparison to the benchmark truth 29](#_Toc526994686)

[6.1: Results on benchmark datasets 29](#_Toc526994687)

[6.2: Mapping accuracy support: 36](#_Toc526994688)

[7- Alignment statistics and the results of AliQC 38](#_Toc526994689)

[7.2 Matches and mismatches, profile of insertions, deletions, transitions and transversions 42](#_Toc526994690)

[7.3 Compatible pairs, reads with unique or multiple alignments 42](#_Toc526994691)

## Bioinformatics

### Magic-BLAST command line parameters

The Magic-BLAST code is available in bin/magicblast. In the simplest situation one may align a fastq RNA-seq file against a reference genome with the command

                bin/magicblast -query run.fastq.gz -subject genome.fasta

a set of recipes is given in the cookbook <https://ncbi.github.io/magicblast> and the complete list of parameters is given by the on-line help:

                bin/magicblast -help

In this analysis Magic-BLAST is invoked via the script Aligners/10_MagicBLAST/align.tcsh.

Magic-BLAST default parameters are optimized for sensitive RNA-seq alignment. Users willing to sacrifice a little sensitivity will see shorter running times and less memory used with these parameters:

-max_db_word_count 10 -limit_lookup T

These parameters will increase filtering of repeated 16-mers in a genome and speed up alignment computation.

Additionally, the default alignment score threshold for reporting reads as aligned is set very low: 20. Setting it higher will result in fewer very short alignments reported. Magic-BLAST scores 1 for each matching pair of bases, and -4 for a mismatch or an indel. To report only alignments that are least 60 bases long with 100% identity or longer alignments with mismatches and indels, use -score 60 option. To report only alignments where at least 70% of read bases match exactly, use -score L,0,0.7 option (where L,b,a means read length * a + b).

To align DNA-seq reads use these parameters:

-splice F -max_db_word_count 10 -limit_lookup T

These parameters will turn off splicing and filter out more repeats. DNA-seq reads are expected to align to more repeats than RNA-seq, so the default filtering will result in a very long run time and high memory use. Higher score thresholds (see above) lead to lower run times when the -splice F option is used.

For large deep sequencing runs (above 10GB) it is typically more efficient to partition the reads into several FASTA or FASTQ files and run several single-threaded Magic-BLAST processes in parallel. The number of partitions should correspond to the number of available CPUs.

### Aligners tested, parameters and performance

#### Aligners tested:

The aligners tested in this study are all open source and public.

##### Magic-BLAST

The latest source code and executables are available from NCBI at

<ftp://ftp.ncbi.nlm.nih.gov/blast/executables/magicblast/LATEST> . In this analysis, we used:

<ftp://ftp.ncbi.nlm.nih.gov/blast/executables/magicblast/1.4.0/ncbi-magicblast-1.4.0-x64-linux.tar.gz>

The magicblast and makeblastdb executables for linux-64 bits were copied into ./bin. The sequence index was constructed using the command

bin/makeblastdb -dbtype nucl -parse_seqids -in myGenome.fasta -out myGenomeDir/genome

##### HISAT2

The HISAT2 (11) code version 2.1.0 was downloaded from <https://ccb.jhu.edu/software/hisat2> .

The sequence index was constructed using the command

bin/hisat2-master/hisat2-build myGenome.fasta myGenomeDir/genome

We ran the make command, moved hisat2-master to the directory ./bin . If our precompiled version of HISAT2 does not execute on your machine, we recommend that you download from <https://ccb.jhu.edu/software/hisat2> your preferred binary or recompile from the source code. Then please move or rsync or link hisat2-master into ./bin.

##### STAR and STARlong

STAR (12,13) was written by Alex Dobin, whom we thank for his feedback. The STAR executables were constructed by downloading the ‘latest release’, i.e. STAR-2.6.0c, from <https://github.com/alexdobin/STAR> in a tmp directory and running the make command (cd STAR/source ; make STAR; clean up; make STARlong), as described in the documentation. The sequence index was constructed using the command

bin/STARlong --runMode genomeGenerate --genomeDir myGenomeDir --genomeFastaFiles myGenome.fasta. The executables were then copied to the bin directory. The STAR 1-pass was run earlier using the previous STAR-2.5.3a version. If our pre

compiled versions of STAR and/or STARlong do not execute on your machine, we recommend that you import STAR again from GitHub, then recompile and move the executables into ./bin.

We checked the release logs at https://github.com/alexdobin/STAR/releases?after=2.6.1b and confirmed that there were no major changes between the versions.

##### TopHat

TopHat (14) is a spliced aligner based on Bowtie and originally written in 2009 by Trapnell C, Pachter L, Salzberg SL. [TopHat: discovering splice junctions with RNA-Seq.](https://www.ncbi.nlm.nih.gov/pubmed/19289445) Bioinformatics. 2009 May 1;25(9):1105-11.

The code was downloaded from <https://ccb.jhu.edu/software/tophat/manual.shtml> by following the instructions. The sequence index was constructed using the command

bin/bowtie2/bowtie2-build myGenome.fasta myGenomeDir/genome

The TopHat and Bowtie2 executables were copied into ./bin.

##### Minimap2

Minimap2 (17) was downloaded from [https://github.com/lh3/minimap2/releases/download/v2.17](https://github.com/lh3/minimap2/releases/download/v2.17/). It works directly with the genome in a FASTA file and does not require a separate genome indexing step.

##### Subread

Subread (18) was downloaded from [http://subread.sourceforge.net](http://subread.sourceforge.net/). The sequence index was constructed using this command:

subread -o myGenome myGenome.fasta

#### Parameters:

Table S1.1: Combinations of aligners and parameters used, the name used for the combination throughout, and the reason for any non-default parameters.

| Name | version | Command line parameters | Reason |
| --- | --- | --- | --- |
| Magic-BLAST | 1.4.0 |  |  |
| STAR 1-pass | 2.5.3a |  |  |
| STAR 2-pass | 2.6.0c | outFilterMatchNmin 24 --outFilterScoreMin 24 --outFilterMismatchNmax 100000 --outFilterMismatchNoverLmax .5  --twopassMode Basic --seedPerReadNmax 100000 | (Dobin and Gingeras, 2016) |
| STAR long | 2.6.0c long | Exact same parameters as STAR 2-pass | Dobin, github FAQ |
| HISAT2 | 2.1.0 |  |  |
| HISAT2 relaxed | 2.1.0 | —minscore L,0.0,-2 | To improve mapping of longer reads |
| TopHat2 | 2.1.1 |  |  |
| Minimap2 | 2.17 | -ax splice -t 6 | For RNA-seq |
| Subread | 1.6.4 |  |  |

Each row lists a name for the program, the non-default parameters (if applicable) as well as a reason for the non-default parameter. The name in column 1 is used throughout the paper to refer to the aligner. Each program takes in addition positional parameters describing the fastQ files to be aligned and the reference genome. These parameters can be found in the master-script (see below).

For Magic-BLAST, STAR 1-pass, HISAT2 and TopHat2, we used the default parameters. The HISAT2 relaxed parameters were tailored to improve the sensitivity of the code on iRefSeq and other long sequences. For STAR, we chose the parameters by reading the documentation, <https://github.com/alexdobin/STAR/blob/master/doc/STARmanual.pdf> , mining the ‘issues’ page on the github page and the RNA-star page at <https://groups.google.com/d/forum/rna-star> . STARlong is recommended for reads over 300 bases. Note that no .GFF annotation file was provided (contrary to the recommendation for STAR 1-pass).

#### Performance:

Table S1.2: Peak memory in GB, for the same experiments for which Figure 10 gives the CPU time in minutes. A “-“ means that we did not perform the run as the aligner is not suitable for the reads. In some cases, we were unable to obtain results and have marked the cell as such.

| **Peak RAM** | Magic-BLAST | HISAT2 | HISAT2 relaxed | STAR 1-pass | STAR 2-pass | STAR long | TopHat2 |
| --- | --- | --- | --- | --- | --- | --- | --- |
| iRefSeq | 16 | 5 | 34 | No results | No results | 42 | No results |
| PacBio | 4 | 5 | 6 | No results | No results | 32 | No resutls |
| PacBio brain | 18 | 5 | 8 | No results | No results | 41 | No results |
| PacBio testes | 19 | 5 | 8 | No resutls | No results | 42 | No results |
| Roche 454 | 14 | 5 | 5 | No results | No results | 32 | 4 |
| Illumina 300+300 | 24 | 5 | 5 | 28 | 32 | 35 | 5 |
| Illumina 250+250 | 21 | 5 | 5 | 28 | 33 | 35 | 5 |
| **Peak RAM** | Magic-BLAST | HISAT2 | HISAT2 relaxed | STAR 1-pass | STAR 2-pass | STAR long | TopHat2 |
| Illumina | 17 | 5 | - | 30 | 32 | - | 5 |
| Human T1 | 26 | 5 | - | 29 | 32 | - | 5 |
| Human T2 | 26 | 5 | - | 29 | 32 | - | 5 |
| Human T3 | 28 | 5 | - | 29 | 32 | - | 5 |
| Malaria T1 | 24 | 0.1 | - | 3 | 4 | - | 3 |
| Malaria T2 | 24 | 0.1 | - | 3 | 4 | - | 3 |
| Malaria T3 | 25 | 0.1 | - | 3 | 4 | - | 3 |
| Human T1 truncated to 50+50 | 21 | 7 | - | 27 | 31 | - | 30 |
| Malaria T1 1% subsampled | 3 | 1 | - | 3 | 6 | - | 1 |

### 1.3 Other Programs

The program ./bin/fastq-dump was used to download the runs from the NCBI Sequence Read Archive, it is available from <https://ncbi.github.io/sra-tools/fastq-dump.html>

The analysis uses several scripts provided in ./scripts and a few executables copied in ./bin. dna2dna and sam2gold are part of the AceView/Magic package. Try ‘dna2dna –help’ and ‘sam2gold –help’ to see the possible options and to check that the programs work on your machine. If you want to recompile or consult the source code , please download the current Magic package from <ftp://ftp.ncbi.nlm.nih.gov/repository/acedb/Software/Magic/Source_code/magic.source.tar.gz> unzip the tar file and recompile in tcsh as follows: ‘setenv ACEDB_MACHINE LINUX_4_OPT ; make libfree.a libtsfree.a libacedna.a ;  cd wacext ;    make sam2gold dna2dna’. The executable will be created in the directory bin.LINUX_4_OPT. You should then copy dna2dna and sam2gold to the ./bin directory. The exact source code used at the time of the analysis can be downloaded from <ftp://ftp.ncbi.nlm.nih.gov/repository/acedb/Software/Magic/archives/Source_code_archives/magic.2018_08_01.source.tar.gz>

- AliQC.py  is a python3 code developed in collaboration with Joe Meehan, FDA, for the MAQC/SEQC project. It was ported to python3 for the present project. The code has several functionalities (try 'python3 AliQC.py --help'), and it is called with the proper parameters by the master script. Its purpose is to scan the SAM files, which can appear indifferently as BAM, SAM, or SAM.gz files, and to extract detailed statistics on the alignments and mismatch patterns. In particular, it reports the cumulated location of the mismatches showing spikes of errors corresponding to specific sequencing cycles. AliQC calls HTSeq (Simon Anders, Paul Theodor Pyl, Wolfgang Huber. HTSeq — A Python framework to work with high-throughput sequencing data.  Bioinformatics 2015 Jan 15; 31(2): 166–169) to parse the SAM files into an object-oriented format. Try ‘python3 AliQC.py –help’. Unfortunately, the configuration of HTSeq cannot always be copied to another machine (because of python) and may need to be reinstalled, a task a little difficult for a biologist but easy for a programmer – see instructions in scripts/HTSeq/README.
- sam2gold: Notice that AliQC.py does not import a truth file, so all the measurements are intrinsic to the SAM file. Comparisons to the 'truth' of the benchmarks is delegated to the C code 'bin/sam2gold'.
- transpose is a simple utility to transpose any tab delimited file.  Usage  ‘cat f1 | scripts/transpose > f2 ‘
- tags is a simple utility to sort and count entries in the first column of a file. Usage ‘tags f1’, in this case the first column ends on the first tab or space, or  ‘tags -t f1’, in this case the first column ends on the first tab
- submit  is a script to control the parallelization of large programs. It is by default configured to allow 4 programs to run in parallel, see line 51, e.g. edit (NCORE = 4) into (NCORE = 8).  By selecting 'farm = local' on line 16, the submissions would become purely sequential. If you have access to a UGE or a SLURM compute-farm, you could submit on the farm by uncommenting line 23 and editing your credentials around line 260. Other farms would be relatively easy to configure. ‘submit’ is called by the main script.

### 1.4 Description of the analysis.

To assess the performances of Magic-BLAST on RNA-seq, we considered several experimental and benchmark runs, aligned them with several aligners and analyzed the resulting SAM files. We provide in GitHub (<https://github.com/ncbi/magicblast>) the programs used in our analysis and a master script, described in section 1.5. Cloning the site will create a scripts directory. The master script can in principle automatically download all the data, run the aligners and repeat the analysis. Of course, running the aligners takes very large resources, so we also provide at <ftp://ftp.ncbi.nlm.nih.gov/blast/demo/magicblast_article/SAM> the pre-computed SAM files. The master script can optionally upload these files rather than realign. The rest of the analysis should proceed correctly in both cases.

The purpose of the paper is to describe the Magic-BLAST aligner and assess its performance, not to distribute an analysis package.  The output of Magic-BLAST is a SAM file. The scripts performing the quality control measures are configured for the selected datasets, for example, they parse the benchmark truth from the custom format chosen by Baruzzo and compare the runs to the truth in a specific manner. The analysis would need to be reconfigured to run on other datasets or on non-Intel/Linux 64 bit machines (in particular because of the Python3 HTSeq library). Contact us if you have problems reproducing our analysis.

### 1.5: How to reproduce this analysis.

It is our hope that one should be able to reproduce our analysis without difficulty on any Linux-64bits machine with 500Gb of disk space and sufficient computing power. Please clone our Github site:

Git clone <https://github.com/ncbi/magicblast>

This will create a subdirectory ./scripts, and a link to the main script MagicBlastPaperMasterScript.tcsh.

cd magicblast/article

tcsh MagicBlastPaperMasterScript.tcsh –help     #  to see the list of the commands and to verify that you can run the script

By running the main script several times with different options, one should successively download the binaries, the data, align the reads and duplicate our analysis as follows:

MagicBlastPaperMasterScript.tcsh init  #  to Initialize the system and download the reference genomes

MagicBlastPaperMasterScript.tcsh download # to download the fasta/fastq files

MagicBlastPaperMasterScript.tcsh sam  # to download the SAM file, or alternatively

MagicBlastPaperMasterScript.tcsh align  # to run the aligners (this takes a lot of time and CPU)

MagicBlastPaperMasterScript.tcsh aliqc # to analyze the results (requires python3 and HTSeq)

MagicBlastPaperMasterScript.tcsh accuracy # to compare to the benchmark truth

MagicBlastPaperMasterScript.tcsh export # To export the final QC tables in RESULTS

You can control the number of runs to be analyzed and the list of aligners by editing the definition of the variable $runs and $methods around line 27 and 50 of the main script MagicBlastPaperMasterScript.tcsh.

The command ‘init’ will download the binaries and the reference genomes from our NCBI ftp site.

The command ‘download’ will import the fasta/fastq files from our ftp site or from SRA. For convenience, we have copied the Baruzzo sequences at our site. We have also copied the Roche fasta file, removing the four-letter barcode from the original SRA entry. The other 3 Illumina and 3 PacBio runs are downloaded directly from the NCBI SRA.  This command will also download the 3 reference genomes used to define the ‘Truth’ of the benchmark: -the iRefSeq are annotated on the human genome GRCh38, limited to the main chromosomes and mitochondrial DNA; -the Baruzzo malaria benchmark is provided with its own reference genome (4), finally -the Baruzzo human data are annotated relative to the hg19 reference genome.

Since computing the alignments is very demanding, two options are provided: one may either download the SAM files from <ftp://ftp.ncbi.nlm.nih.gov/blast/demo/magicblast_article/SAM>  using the command ‘sam’ or rerun the aligners using the command ‘align’. The rest of the analysis will proceed as well in both cases. The main script calls Aligners/*/align.tcsh. These scripts automatically select the datasets fasta/fastq files and the genomic target and construct the genome index specific of each aligner in the directory Aligners/*/$target, where $target is one of (HG19,PFAL,GRCH38). Using these scripts should exactly reproduce our SAM files.

The command ‘aliqc’ will extract the statistics from the sam files.

The command ‘accuracy’ will compare the sam files to the Benchmark truth and report the recall and precision of the introns and the alignments in the directory $method/$run.

Finally, the command ‘export’ will export in the directory RESULTS the quality control as tab delimited tables containing all the numbers that were used to construct the tables and figures in the paper. The construction of the figures, exact layouts/ fonts/ colors, was done manually in excel.

Additional information is provided in the top directory ./README and in bin/README, scripts/README, Aligners/README and scripts/HTSeq/README, but at least in principle, the analysis should be automatic.

## 2-Datasets

### 2.1 Construction of the iRefSeq

The MagicBlastPaperMasterScript automatically downloads the iRefSeq fasta and gff files into the directory ./Fasta/iRefSeq . It also contains all the explanations to construct a new iRefSeq set for a future version of the genome or for a different species. We selected the coding NM_* RefSeq, extracted their gff and used the program ./bin/dna2dna, which reads a gff file and the genome and exports the image of the transcripts by re-synthetizing the RNA using the genomic DNA and the coordinates of the exons.

### 2.2 Description of additional datasets tested

To consolidate our assessment of the aligners’ performance on longer reads and to confirm on much larger datasets the results of the original PacBio run, which was about 40 times shallower (but on average 500 bases longer), two additional PacBio runs were added to the study: the **PacBio** **testes SRR5189667** and **PacBio brain SRR5189652**, studied in Lagarde et al (14). Noticeably, these PacBio runs have a much lower level of mismatches than our original ‘PacBio’ from fetal lung, because Lagarde et al ‘corrected’ the PacBio sequences by using for each dataset a parallel deep Illumina run done on the same RNA sample. We hope this transformation of the RNA-seq sequences was conservative and careful, and regret that the original raw PacBio data was not made available also in SRA.

The results on the Illumina run did not match well the results on the human T1 Baruzzo benchmark, and we needed to distinguish the impact of the depth of coverage (10 times larger in Illumina than in the benchmark) and of the data type, experimental or simulated. As a first step, we subsampled the Baruzzo malaria T1r1 set to the same coverage as the Human Baruzzo T1r1 set: we took 1 read pair every 100 exactly: this dataset appears as **Malaria T1r1S 1% subsample** (for T1r1 Subsampled).

We also wanted to test impact of shorter Illumina reads, as many SRA runs are 50+50 bases, and we tested a copy of **Baruzzo human** **T1 r1_**50 truncated to 50+50, in which we used only the first 50 bases of each read (50+50).

Finally, Magic-BLAST performance as an aligner is excellent for very long reads (Roche, PacBio or iRefSeq) but not as good for Illumina 101+101, or the Baruzzo benchmark (100+100), where STAR and HISAT in this order align faster and better. The trend for Illumina has been to extend the read pairs to 150+150 (standard on X10) or even 250+250 or 300+300. To better compare the performance of Magic-BLAST and STAR (long or regular) on Illumina longer reads, we added two recent Illumina runs with 250+250 and 300+300 paired end reads. The long Illumina read pairs **Illumina 300+300** **SRR5437876, from MCF7 cells**, were studied in (1) and generated by ’Roche sequencing solutions’. The **Illumina 250+250 PE** **SRR5438850** is a 23.3million fragments from metastatic melanoma.

All the results for the original and additional runs can be found in supplementary tables 2,3,4,7,8.

### 2.3 Histograms of length to be aligned and effectively aligned by each program

The data are in Table S2 in the excel book, which provides support for Figure 3, 8, and S2.1 to S2.16. In this analysis, each multiply aligned read is counted only once, at the location of its BAM primary alignment (excluding the secondary alignments with flag 256). To facilitate reuse of the excel tables, which are usually very large, we provide for each table an index listing the content of the columns and lines, in the order in which they appear in the large excel table. Table 2 has 140 columns, listed in Table S2.1 index below in the order they appear in the table. Each column gives the histogram of length for each combination sample x method listed. Each line is a bin of variable size, as indicated. The whole table has 903 lines, which correspond to the number of 100-bases bins in iRefSeq. Most datasets have much fewer bins (e.g. 100 bins for Baruzzo sets).

Table S2.1: index of content and organization of excel Table S2.

| **Sample** | **histograms of length appear in this order in Table S2, one column per histogram** |
| --- | --- |
| Actual read in each sample, all in bins of 100 bases | iRefSeq, PacBio, Roche 454 (1/100), Illumina pairs 101+101(1/50000), PacBio Brain SRR5189652 (1/20), PacBio Testes SRR5189667 (1/20) |
| # iRefSeq (bin 100 bases) | Actual reads, Magic-BLAST, HISAT2 relaxed, HISAT2, STAR 1-pass, STAR long, STAR 2-pass, TopHat2 |
| # PacBio (bin 30 bases) | Actual reads, Magic-BLAST, HISAT2 relaxed, HISAT2, STAR 1-pass, STAR long, STAR 2-pass, TopHat2 |
| # Roche (bin 10 bases) | Actual reads, Magic-BLAST, HISAT2 relaxed, HISAT2, STAR 1-pass, STAR long, STAR 2-pass, TopHat2 |
| # Illumina 101 bases (Bin 1) | Actual reads, Magic-BLAST, HISAT2, STAR 1-pass, STAR 2-pass, TopHat2 |
| # SRR5189652 PacBio brain (bin 30) | Actual reads, Magic-BLAST, HISAT2 relaxed, HISAT2, STAR long, TopHat2 |
| # SRR5189667 PacBio testes (bin 30) | Actual reads, Magic-BLAST, HISAT2 relaxed, HISAT2, STAR long, STAR 2-pass, TopHat2 |
| # SRR5438850 (Illumina 250 bin 30) | Actual reads, Magic-BLAST, HISAT2, STAR long, STAR 2-pass |
| # SRR5437876 (Illumina 300 bin 30) | Actual reads, Magic-BLAST, HISAT2, STAR long, STAR 2-pass |
| # Human_T1 (bin 1) | Actual reads, Magic-BLAST, HISAT2, STAR 1-pass, STAR long, STAR 2-pass, TopHat2 |
| # Human_T2 (bin 1) | Actual reads, Magic-BLAST, HISAT2, STAR 1-pass, STAR long, STAR 2-pass, TopHat2 |
| # Human_T3 (bin 1) | Actual reads, Magic-BLAST, HISAT2, STAR 1-pass, STAR long, STAR 2-pass, TopHat2 |
| # Malaria_T1 (bin 1) | Actual reads, Magic-BLAST, HISAT2, STAR 1-pass, STAR long, STAR 2-pass, TopHat2 |
| # Malaria_T2 (bin 1) | Actual reads, Magic-BLAST, HISAT2, STAR 1-pass, STAR long, STAR 2-pass, TopHat2 |
| # Malaria_T3 (bin 1) | Actual reads, Magic-BLAST, HISAT2, STAR 1-pass, STAR long, STAR 2-pass, TopHat2 |

Figure S2.1 Actual read length distributions for experimental sets as well as iRefSeq. Here we show, on top of the datasets in Figure 3, the length distribution for two PacBio runs from real tissues used in (14) by Lagarde et al. to annotate long non-coding transcripts. The average read length from each dataset is indicated. The scale in y is reduced 100 times for Roche 454, 50000 times for Illumina, and 20 times for two additional PacBio runs.

Figure S2.2 a to d show the actual and mapped length histograms for the two additional PacBio runs (15), and then for the two long illumina runs. For Illumina, the plot shows on the right a zoom in on the last bin (quasi fully or fully aligned). The sharp peak at 30 bases in the HISAT2 relaxed and the Magic-BLAST alignments corresponds to a partial match of the PacBio adaptor to the genome.

S2.2 S2.3

Figure S2-3a to f show the length histogram for the Baruzzo benchmark runs, in logarithmic scale, then the associated Figures S2.3a’ to f’ focus on point 100 bases and show the number of reads aligned over 100/100 bases by each aligner. TopHat2 alignments are all quasi complete: only alignments 98 bases or longer are allowed by default. Similarly, HISAT2 accepts only alignments of 90 bases at least. The three versions of STAR (1 pass or 2-pass or STAR long) accept aligned fragments of 30 to 35 at least. Magic-BLAST accepts alignments down to 20 bases, likely taking the risk of getting some non-specific mapping, or even some mis-mappings. These plots clearly show the problem posed to TopHat2 and to HISAT2 by increased levels of mismatches. Magic-BLAST resists very well to very high levels of mismatches (T3 levels), STAR copes slightly less gracefully, yet yields more complete alignments than Magic-BLAST.

## 3-Introns discovered, per coverage (ROC curves)

### 3.1 Supporting data for the ROC curves:

All numbers supporting the ROC curves are in the excel sheet Table S3, which is a very large table. We detail in this paragraph the layout and content of this table. An alignment supporting an intron is defined by a line in the SAM/BAM file where the CIGAR contains an N with minimal intron length 50 bases. When a read is aligned at multiple sites, each of its alignments supporting an intron is counted. Some spliced genes are truly repeated, some are very similar. If one rejects all reads multiply aligned, the introns of repeated genes cannot be detected, therefore, we kept the introns detected by multiply aligned reads. This allowed us not to artificially overestimate the specificity of methods unable to select the true best position. Note that in the benchmark, all reads are attributed to unique positions even when they exactly align in multiple sites. Also, a single read may support several neighboring introns.

Table S3 and S4 report intron discovery and have twelve columns, listed below in Table S3.1:

Table S3.1: Content of the columns in excel Tables S3 and S4

| Column 1 | Species |
| --- | --- |
| 2 | Run (dataset) |
| 3 | Method (Aligner and parameters) |
| 4 | Minimal Intron support |
| 5 | Intron in benchmark |
| 6 | Intron discovered in method |
| 7 | FP: False positive Intron |
| 8 | TP: True positive Intron |
| 9 | FN: False negative Intron |
| 10 | Intron discovery precision p=TP/(TP+FP) |
| 11 | Intron discovery recall r=TP/(TP+FN) |
| 12 | Intron discovery F-score 2pr/(p+r) |

Excel Tables S3 and S4 show results for all 24 datasets and include all 18 runs from Baruzzo (6 times 3 as each run came in triplicate), the two modified Baruzzo, truncated to 50 bases and subsampled 1 in 100. They also include all experimental runs tested (3 PacBio, 1 Roche, 3 Illumina (300, 250 and 101 bases paired end reads) and iRefSeq . The runs appear in the order below (Table S3.2); for each run the methods indicated are reported successively. Excel Table S3 shows the number of introns (true, false or missed) with at least 1, 2, 3... 100 supporting reads. In some cases, a method does not find any intron with 100 supports, and the table stops as soon as no introns are found above the indicated level. This top ‘minimal intron support’ level is indicated in column 4 of Table S3 and S3.3, for all methods (in the same order as for the method’s column 3). For instance, for iRefSeq, where the most supported intron in the annotation is supported by 51 alternative variants hence has 51 support, the iRefSeq line in column 4 reads (51,75,75,43,1), which means that Magic-BLAST top supported intron also has 51 support (we have not checked that they are the exact same as in iRefSeq truth), HISAT2 relaxed or regular top intron has 75 support (showing that this top intron received at least 50% support that did not belong there), and STAR long top intron has 43 support, showing at least 8 missed support. The regular STAR 2-pass has very few alignments and its introns are supported by only 1 read per NM transcript.

| Species | Run | Methods (in order of appearance in the table) | top minimal support recorded: from 1 to the number indicated below, for each method, in order |
| --- | --- | --- | --- |
| Human | HG19t1r1 | 10_MagicBLAST,21_HISAT2,30_STAR,31_STARlong,32_STAR.2.6c,40_TopHat2 | 100,100,100,100,100,100 |
| Human | HG19t1r1_truncate50 | 10_MagicBLAST,21_HISAT2,30_STAR,31_STARlong,32_STAR.2.6c,40_TopHat2 | 62,100,98,100,100,100 |
| Human | HG19t1r2 | 10_MagicBLAST,21_HISAT2,30_STAR,31_STARlong,32_STAR.2.6c,40_TopHat2 | 100,100,100,100,100,100 |
| Human | HG19t1r3 | 10_MagicBLAST,21_HISAT2,30_STAR,31_STARlong,32_STAR.2.6c,40_TopHat2 | 100,100,100,100,100,100 |
| Human | HG19t2r1 | 10_MagicBLAST,21_HISAT2,30_STAR,31_STARlong,32_STAR.2.6c,40_TopHat2 | 100,100,100,100,100,100 |
| Human | HG19t2r2 | 10_MagicBLAST,21_HISAT2,30_STAR,31_STARlong,32_STAR.2.6c,40_TopHat2 | 100,100,100,100,100,100 |
| Human | HG19t2r3 | 10_MagicBLAST,21_HISAT2,30_STAR,31_STARlong,32_STAR.2.6c,40_TopHat2 | 100,100,100,100,100,100 |
| Human | HG19t3r1 | 10_MagicBLAST,21_HISAT2,30_STAR,31_STARlong,32_STAR.2.6c,40_TopHat2 | 96,52,100,100,100,64 |
| Human | HG19t3r2 | 10_MagicBLAST,21_HISAT2,30_STAR,31_STARlong,32_STAR.2.6c,40_TopHat2 | 100,51,100,100,100,75 |
| Human | HG19t3r3 | 10_MagicBLAST,21_HISAT2,30_STAR,31_STARlong,32_STAR.2.6c,40_TopHat2 | 100,51,91,100,100,38 |
| Human | Illumina | 10_MagicBLAST,21_HISAT2,30_STAR,32_STAR.2.6c,40_TopHat2 | 100,100,100,100,100 |
| Human | PacBio | 10_MagicBLAST,20_HISAT2_relaxed,21_HISAT2,31_STARlong | 100,100,76,100 |
| Human | Roche | 10_MagicBLAST,20_HISAT2_relaxed,21_HISAT2,31_STARlong,32_STAR.2.6c,40_TopHat2 | 100,100,100,100,17,100 |
| Human | SRR5189652 | 10_MagicBLAST,20_HISAT2_relaxed,21_HISAT2,31_STARlong,32_STAR.2.6c | 100,100,100,100,1 |
| Human | SRR5189667 | 10_MagicBLAST,20_HISAT2_relaxed,21_HISAT2,31_STARlong,32_STAR.2.6c | 100,100,100,100,1 |
| Human | SRR5437876 | 10_MagicBLAST,21_HISAT2,31_STARlong,32_STAR.2.6c | 100,100,100,100 |
| Human | SRR5438850 | 10_MagicBLAST,21_HISAT2,31_STARlong,32_STAR.2.6c | 100,100,100,100 |
| Human | iRefSeq | 10_MagicBLAST,20_HISAT2_relaxed,21_HISAT2,31_STARlong,32_STAR.2.6c | 51,75,75,43,1 |
| Malaria | PFALt1r1 | 10_MagicBLAST,21_HISAT2,30_STAR,31_STARlong,32_STAR.2.6c,40_TopHat2 | 100,100,100,100,100,100 |
| Malaria | PFALt1r1Subsampled | 10_MagicBLAST,21_HISAT2,30_STAR,31_STARlong,32_STAR.2.6c,40_TopHat2 | 13,18,16,17,20,18 |
| Malaria | PFALt1r2 | 10_MagicBLAST,21_HISAT2,30_STAR,31_STARlong,32_STAR.2.6c,40_TopHat2 | 100,100,100,100,100,100 |
| Malaria | PFALt1r3 | 10_MagicBLAST,21_HISAT2,30_STAR,31_STARlong,32_STAR.2.6c,40_TopHat2 | 100,100,100,100,100,100 |
| Malaria | PFALt2r1 | 10_MagicBLAST,21_HISAT2,30_STAR,31_STARlong,32_STAR.2.6c,40_TopHat2 | 100,100,100,100,100,100 |
| Malaria | PFALt2r2 | 10_MagicBLAST,21_HISAT2,30_STAR,31_STARlong,32_STAR.2.6c,40_TopHat2 | 100,100,100,100,100,100 |
| Malaria | PFALt2r3 | 10_MagicBLAST,21_HISAT2,30_STAR,31_STARlong,32_STAR.2.6c,40_TopHat2 | 100,100,100,100,100,100 |
| Malaria | PFALt3r1 | 10_MagicBLAST,21_HISAT2,30_STAR,31_STARlong,32_STAR.2.6c,40_TopHat2 | 100,100,100,100,100,100 |
| Malaria | PFALt3r2 | 10_MagicBLAST,21_HISAT2,30_STAR,31_STARlong,32_STAR.2.6c,40_TopHat2 | 100,100,100,100,100,100 |
| Malaria | PFALt3r3 | 10_MagicBLAST,21_HISAT2,30_STAR,31_STARlong,32_STAR.2.6c,40_TopHat2 | 100,100,100,100,100,100 |

Table S3.2: index of methods tested on each dataset, and the order in which they appear (from top to bottom) in Excel Tables S3 and S4. The last column shows the minimal number of supporting reads, this is the number of lines in table S3 (excel) for the particular (sample x aligner) combination. If this number is 100, there were some introns supported by 100 reads or more. If this number is say, 52, like in Baruzzo human T3r1 aligned with HISAT2, then the maximal number of support observed was 52 in HISAT2 and Table S3 shows only 52 lines, not 100, for that particular combination.

### 3.2 Graphical representations for additional runs and zoomed views:

Table S3 has close to 14400 lines with values. The precision, recall and F-score for intron discovery are computed at each minimal support value. A graphical representation is given in Figure 4 and supplementary figures S3.1 to S3.10: the ROC plots show, for each dataset and aligner, the false positive (or unannotated in iRefSeq if there is no truth) in x and the true positive (or annotated in iRefSeq) in y.

Figure S3.1

Figure S3.1 Zoom on the ROC curve for introns discovery (by coverage from 100 to 1, truncated) for the Illumina run. STAR 1-pass (dotted green) is just below Magic-BLAST for coverages above 25 and would be an acceptable second choice, but this is not the case for STAR 2-pass, which has many more introns supported by at least 100 reads yet unannotated. (red: Magic-BLAST, dotted green: STAR 1-pass, bright green continuous line: STAR 2-pass, light blue and gray: TopHat, Yellow-orange: HISAT2)

Figure S3.2, S3.3, S3.4, S3.5: Global view of the ROC curve for introns discovery in the ultra-deep malaria sets and in the one in one hundred subsampled malaria T1 set. Coverage from 100 supporting reads to 1; color code same as in figure 4 (Magic-BLAST red, HISAT2 yellow, Star 1-pass dotted line light green, STAR 2 pass continuous line light green, STAR long dark green). At low coverage, STAR has an immense number of false positives, STAR long 2-pass is intermediate between STAR 2-pass and STAR 1-pass. On malaria T1, STAR 1-pass is much more precise than STAR 2-pass, as observed in Figure 4f. 1/100 subsampling of malaria T1 in figure S3.3 shows that this effect vanishes when the amount of data is reduced to about the same coverage as in the human T1 dataset, reinforcing the idea that the second step in STAR tends to reinforce and validate the erroneous introns found in the first step.

#### New datasets

Figure S3.6 shows that, when reads are truncated from 100+100 bases to 50+50 bases in human T1, Magic-BLAST remains the most precise, but loses too much on recall, so altogether it no longer has the lead for intron discovery in 50+50 bases runs. HISAT2 is top and left and in the most desirable position (for 2 supports or more), followed (surprisingly) by the STAR long program. The regular STAR is less precise and less sensitive on 50-mer, with a slight advantage to the 2-pass version for low coverage introns. TopHat is extremely imprecise and has many false positive at very high coverage. There are 137919 true introns in human T1 R1.

Figure S3.7 to S3.10 (below) show that Magic-BLAST has the best ROC curves for the two additional PacBio and long Illumina runs.

## 4- Intron precision and recall

The statistics for intron discovery with any support (minimum = 1) and at best F-score (hence at a coverage variable by dataset and aligner) were extracted from the master Table S3 and copied into the twin tables S4 (which support some columns of figures 5 and 6). The twin Tables S4 give the numbers of TP, FP and FN introns supporting the precision, recall and F-score. They have the same 12 columns as table S3 and have 159 lines.

### All experimental datasets and iRefSeq

In complement to Figure 5 (columns a,b,c) which show the precision, recall and F-score for all introns in the 3 experimental sets and iRefSeq, Figure S4.1 and S4.2 extend this presentation to all real datasets, adding the two PacBio and two long Illumina runs, and show precision, recall and F-score for all introns (minimum coverage 1, Figure S4.1) and at the coverage optimizing the F-score (S4.2).

For all introns (S4.1), Magic-BLAST has the highest F-score in all experiments deposited in SRA and in iRefSeq, except for Illumina 101+101, where its F-score is 1.5% below TopHat, yet on this sample it wins over Star 2-pass by 20% and HISAT2 by 23% because these two programs have very low precision (TopHat has 2.8% better precision than Magic-BLAST on this particular dataset). Magic-BLAST F-score surpasses all competitors by a margin of 2 to 4% for the six runs from any technology whose length is 300 bases or more (Illumina paired end 300+300, Roche, the three PacBio sets, edited or not, or the iRefSeq benchmark). For Illumina 250+250 bases, Magic-BLAST is still the best but STAR long follows 0.4% away.

Concerning the precision of intron discovery, Magic-BLAST is usually the best, the exceptions are the PacBio testes sample, where HISAT2 win in precision by 15% but is calling six times less introns, and Roche and Illumina where TopHat has an edge of 4 or 3% on precision but finds 41% less introns in Roche and 2% less in Illumina. Finally, on the long Illumina runs, Magic-BLAST and STAR-long fight for the first position, Magic-BLAST being 3% more precise on the 300+300 while STAR long is 0.8% more precise on the 250+250 set. [we ignore STAR 2-pass on the 3 PacBio or the Roche, which discovered just a few introns].

Examining now Figure S4.2, which shows precision, recall and F-score for introns called at the coverage optimizing the F-score, another interesting number here is the read coverage at which an aligner produces the best F-score. This number is written in parenthesis after the name of the program. For the shallow human sets, this number is 1 or 2 for PacBio and Roche, or 3 or 4 for the long Illumina runs. But for the deepest experimental Illumina set at 101+101 bases, interestingly, all programs achieve their best F-score at significant read coverage. Magic-BLAST has the lowest, with seven, and HISAT2 requires 20, TopHat2 12 and STAR 10. The malaria ultra-deep sets (Figure S4.6) also see HISAT2 and STAR achieving their best F-score at coverages that range from 4 to 13 (Table S4).

Figure S4.1: precision, recall and F-score for all introns in experimental datasets.

Figure S4.2: Precision, recall and F-score for introns called at the coverage optimizing the F-score in the experimental datasets.

### The Human Baruzzo benchmark (100+100) and the human T1 run truncated at 50+50

Figure S4.3 shows the intron discovery precision, recall and F-score at any coverage, for all nine samples of the human benchmark, including the run T1 r1 where all reads have been truncated at 50+50 bases (taking the first 50 bases of both reads in each fragment. Figure S4.4 shows the same but at the best F-score, which is reached at coverage 1 for all T1 samples, including the truncated at 50 bases, or 2 or more, where indicated in parenthesis behind the program name in the legend of the precision plot. HISAT2 and STAR often attain their best F-score at coverage 2 in this shallow human dataset in T2 and T3 samples, where the levels of mismatches are 1.2% and 6%, respectively. Notice that the three simulated runs, for each level of mismatches T1, T2 or T3, give extremely similar results. This is the reason why we showed only run r1 in all plots and tables (like Baruzzo did in (4)).

In this Illumina-like human benchmark, Magic-BLAST is the best in all cases, it has the best F-score in all cases, except the T1 sample where all reads were truncated from 100+100 paired end reads to 50+50. In this sample, precision remains the best, 3.4% above STAR 2-pass and 6% above HISAT2, but the recall lags by 13% relative to HISAT2 and 10% relative to STAR. That is because, as reads become shorter and closer to the seed length, it is more difficult to seed and extend, but the introns found remain highly specific, only 0.2% less precise in the 50+50 version than in the 100+100. Magic-BLAST indeed has the best precision by a margin in all cases (if we ignore the T3 runs, where TopHat aligns very little but with good precision).

### 4.3 The malaria Baruzzo benchmark and the 1/100 subsampled malaria run

Since runs r1, r2 r3 are so similar, we show in Figure S4.5 below the view with only runs r1 for the T1 T2 and T3 levels of mismatches or SNVs, in malaria and human. For convenient comparison of the shallow human and deep malaria benchmarks, we added two *in silico* experiments where

- We evaluated the impact of read length on intron finding, when using relatively short Illumina reads. We truncated all reads from 100 bases to 50 bases in Human T1 r1: T1 mimics good Illumina 100+100 paired ends on good genome, the truncated T1 mimics good Illumina 50+50 paired ends on good genome. We acted on the human set because it has close to 200000 introns to evaluate while malaria has 40 times less (only about 5000).
- We examined the impact of depth of coverage on intron discovery, by subsampling and taking 1 in 100 read pairs in the malaria sample T1 r1, taking 1 skipping 99 in the original run. This subsampling puts the malaria sample at approximately the same coverage as the human T1 sample.

The benchmark sets are useful to evaluate the performance of the aligners on typical 100+100 bases paired end reads *a la* Illumina. As we showed before, the comfort zone of Magic-BLAST is for longer reads, larger than say 140 bases: there it would unambiguously be the top choice since it excels both at mapping and at intron discovery, and this is true for both perfectly matching and highly mismatched (like unedited PacBio) datasets. But when applied to the Illumina-like benchmark, Magic-BLAST has some strengths as well.

Results for the human have been discussed above, but let us summarize: the F-score for intron discovery puts Magic-BLAST in first position in T1 T2 and T3; the worse the quality, the more it gains on other aligners: in T1, it is 0.2% better than HISAT and 1.2% above STAR 2-pass; in T2 it is 2.3% better than HISAT and 3.5% above STAR 2-pass and in T3, where HISAT stops aligning, it is 7.3% above STAR 2-pass. It does not have the best recall, but it wins because it has much better precision. Its introns are more trustable.

Results for malaria show again that Magic-BLAST has the best F-score at intron discovery in T1 T2 T3, with TopHat the second best in T1 and T2. HISAT is behaving much better than STAR on malaria, still very far behind Magic-BLAST by all criteria. In T1, Magic-BLAST wins over TopHat by 3%, HISAT2 by 15% and STAR 2-pass by 58%; in T2 it wins over TopHat by 6%, HISAT2 by 27% and STAR 2-pass by 79%, in T3 it wins over HISAT2 by 22%, TopHat by 32% and STAR 2-pass by 76%. Indeed, in contrast to human, malaria causes a huge difficulty for STAR, and to a lesser extent for HISAT as well: their precision drops dramatically while the recall, for such ultra-deep experiments, remains in the range 88-96% for T1 T2. In T3 malaria, the recall drops considerably in TopHat and HISAT; recall for STAR 2-pass is 2.3% above magic-BLAST but its precision is abysmal at 2.3% versus 78% for Magic-BLAST.

The subsampling experiment we performed on the malaria sample T1 was very informative: only about 54% of the introns still exist in the 100 times smaller dataset and recall went from 93 to 96% in T1 to 42-54% in the subsampled set. But the most striking observation is how precision dramatically increased for STAR (+65%) and HISAT (+26%). This is true also for the STAR 1-pass. This indicates a built in problem evident on ultra-deep runs (in malaria) that will lead these two programs to generate junk introns when the datasets are deep enough. This feature means that increasing the size of an RNA-seq experiment will adversely affect the quality of the introns discovered, very strongly by STAR and strongly by HISAT. Magic-BLAST is not affected by this curse! It might be nice to eliminate the possible effect of the genome composition, and to test a 100 times larger human simulated set a la Baruzzo, but we did not yet succeed in generating such a set.

Magic-BLAST retains the best precision in the subsampled experiment, at 99.7%, however at this low coverage it has 5% less recall than STAR and HISAT and its F-score is below HISAT by 3.6% and below STAR 2-pass by 2%. This is the one and only exception, related to the very shallow nature of this dataset (100 000 paired reads).

Our program is clearly the best in all situations and the most equilibrated, gracefully coping with all the variables in the Baruzzo benchmark.

Figure S4.5: Precision, recall and F-score for introns called in the Baruzzo benchmark and in two related experiments: read truncation and 1/100 run subsampling.

## 5- Results on the iRefSeq dataset

Table S5 shows the statistics graphically represented in Figure 7 of the article.

|  |  | iRefSeq Truth | Magic-BLAST | HISAT2 relaxed | HISAT2 | STAR long | TopHat2 |
| --- | --- | --- | --- | --- | --- | --- | --- |
| Alignment statistics | iRefSeq aligned | 45108 | **45108** | **45108** | 40445 | 37171 | 0 |
|  | Bases aligned | 1.55E+08 | **1.53E+08** | 1.45E+08 | 1.37E+08 | 1.11E+08 | 0 |
|  | % iRefSeq aligned | 100% | **100%** | **100%** | 89.66% | 82.40% | 0% |
|  | % bases aligned | 100% | **99.04%** | 94.04% | 89.50% | 82.37% | 0% |
|  | Bases mismatched | 0 | **771** | 66358 | 87963 | 38018 |  |
|  | Mismatches per kb aligned | 0 | **0.005** | 0.456 | 0.643 | 0.344 |  |
|  | iRefSeq with multiple alignments | 229 | 294 | 1941 | 733 | **223** |  |
|  | % iRefSeq with multiple alignments | 0.51% | 0.65% | 4.30% | 1.81% | **0.60%** |  |
|  |  |  |  |  |  |  |  |
| Mapping accuracy | Perfect alignment, complete, no mismatch (TP1) | 45108 | **43826** | 39757 | 37945 | 36342 | 0 |
|  | Partial alignment, no mismatch (TP2) | 0 | 1086 | 4256 | 819 | **58** | 0 |
|  | Partial or complete misalignment, has mismatches (FP) | 0 | **196** | 1095 | 1681 | 829 | 0 |
|  | Unmapped (FN) | 0 | **0** | **0** | 4663 | 7937 | 45108 |
|  |  |  |  |  |  |  |  |
|  | % perfect | 100% | **97.16%** | 88.14% | 84.12% | 80.57% | 0% |
|  | % partial | 0% | 2.41% | 9.44% | 1.82% | **0.13%** | 0% |
|  | %misaligned | 0% | **0.43%** | 2.43% | 3.73% | 1.84% | 0% |
|  | %unmapped | 0% | **0.00%** | **0.00%** | 10.34% | 17.60% | 100% |
|  |  |  |  |  |  |  |  |
|  | Mapping precision | 100% | **99.57%** | 97.57% | 95.84% | 97.77% |  |
|  | Mapping recall | 100% | **100.00%** | **100.00%** | 89.26% | 82.10% | 0% |
|  | Mapping F-score | 100% | **99.78%** | 98.77% | 92.44% | 89.25% |  |
| Special cases of | Wild misalignment, at a genomic site not overlapping the truth |  | **12** | 103 | 39 | 100 |  |
| misalignment | Overlapping the truth but extending outside, creating a new and incorrect first or last exon (and by inference incorrect promoters or 3'ends) |  | **5** | 191 | 79 | 58 |  |
|  |  |  |  |  |  |  |  |
| Introns | True Positive: introns correctly found | 210509 | **206850** | 201854 | 197249 | 124993 | 0 |
|  | False Negative: missed introns | 0 | **3659** | 8655 | 13260 | 85516 | 210509 |
|  | False Positive: invented introns | 0 | **158** | 2336 | 2765 | 619 | 0 |
|  |  |  |  |  |  |  |  |
|  | Intron discovery precision | 100% | **99.92%** | 98.86% | 98.62% | 99.51% |  |
|  | Intron discovery recall | 100% | **98.26%** | 95.89% | 93.70% | 59.38% | 0% |
|  | Intron discovery F-score | 100% | **99.09%** | 97.35% | 96.10% | 74.37% | 0% |

Repeated genes in iRefSeq: Examples accessions are NM_001080142 (nine copies CT47A gene family), NM_001242327 (seven copies USP17L family), NM_001130406 (5 copies of PRR20D family). Counter-examples that seem to be repeated but are not: NM_000176 or NM_001204258-4264, which represent 8 transcripts that are all identical in sequence, yet they belong to a single gene NR3C1 but are annotated with different initiation sites…

## 6- Mapping accuracy by comparison to the benchmark truth

### 6.1: Results on benchmark datasets

Excel Table S6 (excel sheet) evaluates the accuracy of the mapping by comparing in each method, read by read, the coordinates of the ends of its alignments to the unique reference alignment provided by the Baruzzo benchmark [Nature Methods 14.2 pp135-139, 2017]. Each read is aligned uniquely in the benchmark.

In each method, the aligner may recover the true chromosomal coordinates of the first and last base of the read (TP1), or provide a partial alignment included in the true segment (TP2) or overlap the true position but extend out of it (FP1), or align completely elsewhere (FP2), or fail to align (FN).

The first half of the table (columns 10 to 20) counts reads: if a read aligns at several sites, it counts as exact (or partial) if at least one of its alignments has the same coordinates as the benchmark unique alignment (or is included). In contrast, the second half of the table, columns 22 to 32, reports on alignments: each alignment is counted in its category. This is a more accurate measure of alignment accuracy since keeping too many ambiguous mappings is penalized. The precision (p=(TP1+TP2)/(TP1+TP2+FP1+FP2)), recall (r=(TP1+TP2)/(TP1+TP2+FN)) and F score (f=2 p r/(p+r)) are computed for reads and alignments.

This type of analysis can only be applied to datasets where a mapping ‘truth’ is available, i.e. to the Baruzzo and iRefSeq benchmarks. Note that in Baruzzo, the ‘truth’ is coarse and incomplete, since no effort was made to provide multiple mappings of equal quality when they exist. We even found tens of thousands of reads that upon addition of mismatches by the generative tool of Baruzzo, end up mapping exactly without mismatch at another genomic site: a good aligner will move them away from the original site (TP) to a false positive site (FP2). However, these systematic errors will not prevent meaningful comparison of the aligners, since all will suffer equally from the problem. This and the short size of the reads (100 bases) explains why we chose a coarse measure, dependent only on the end coordinates, in the Baruzzo sets. However, in the case of iRefSeq, where the read length and the number of exons is often enormous and where truth is more curated, we applied in Table S5 a more precise treatment, calling true positive (TP) and false positives (FP) by using, in addition to the match of end coordinates, the number of matching bases relative to the truth and the presence of mismatches.

Figure S6.1 shows the number of alignments of the four types that represent a partition of the data: True positive complete alignments (with exact match of end coordinates, dark green), True Positive partial alignments (where coordinates of the alignment are within the coordinates of the truth, light green), misalignments (incorrect placement, red), or number of reads missed alignments (black). Each dataset has 20 million reads (except for the malaria T1 1% subsampling). The number of alignments is usually larger, because some reads have multiple alignments (Figure S6.2), and a single read may have multiple partial alignments, as observed in TopHat on the human T1 dataset truncated at 50 bases. The number of unaligned reads (black) is especially large in HISAT and TOPHAT in the presence of high mismatch rate, T3 and T2. For good quality data (T1) all programs align most reads at the correct position, although in the truncated dataset T1 cleaved at 50+50 bases, Magic-BLAST is missing more cases. It also has, in all human datasets, a larger number of misalignments. But this defect is observed only with reads up to 100 bases long and vanishes for longer Illumina reads. Indeed, in 250+250 or 300+300 Illumina runs, as well as in Roche and Pacbio, Magic-BLAST is considerably more efficient than the other aligners tested (Figure S7.1 below).

Figure S6.1bis is the same data as figure S6.1 but now in percentage of all alignments. Figure S6.1ter copies the data from S6.1 focusing on the T1 sample. The number of alignments of each category is plotted; the percentage of all alignments plus unmapped reads is listed on each category, so that this plot gives the same information as figure 6c for the iRefSeq. Comparison between Figure 6c and S6.1ter exemplifies how magic-BLAST takes advantage of extended read length to improve its accuracy.

The misalignments (red in Figure S6.1) consist of two categories, the bulk where coordinates of the alignment do not overlap coordinates of the truth, and a minor category where the alignment overlaps the truth but extends beyond the true segment (Figure S6.3). This category poses a serious problem for gene reconstruction because it may predict incorrect exons or incorrect extension of the gene upstream or downstream. The number of reads in this latter category is shown in Figure S6.3: in all datasets, Magic-BLAST has the smallest number of defects in this category. This desirable property correlates with the fact that Magic-BLAST also has the largest number of partial alignments (Figure S6.4). In case of doubt, Magic-BLAST prefers to clip an alignment, therefore generating a partial rather than force an extended alignment not strongly supported by the sequence.

Figure S6.5 Alignment accuracy, precision, recall and F-score for all methods and datasets tested.

### 6.2: Mapping accuracy support:

All numbers for this analysis are in Excel Table S6, which has 30 columns described in self-explanatory terms in Table 6.1. Columns 10 -20 (and 22-32) respectively show the number of TP FP FN reads (and alignments) of the various types and the read (and alignment) mapping precision recall and F-score. Other columns in the table bring a variety of information on unique and multiply mapped reads, on partials and the detailed types of congruence to the mapping. Figure 6 displays the alignment precision and recall.

Table S6.1: Content of columns in Excel Table S6.

| Column 1 | Species |
| --- | --- |
| 2 | Run |
| 3 | Method |
| 4 | Reads in benchmark |
| 5 | Reads aligned in method |
| 6 | Reads uniquely aligned, U |
| 7 | Reads multi-aligned, NU |
| 8 | % reads multiply aligned NU/(U+NU) |
| (9) | READS |
| 10 | Misaligned reads some partial, FP=FP1+FP2 |
| 11 | Overlapping the truth, but extending out, FP1 |
| 12 | Mis-mapped reads not overlapping the truth, FP2 |
| 13 | Reads at exact same site as benchmark, TP1 |
| 14 | Read at correct site, but partial, TP2 |
| 15 | Correct mapping, some partial, TP=TP1+TP2 |
| 16 | Unaligned FN |
| 17 | Read mapping precision TP/(TP+FP) |
| 18 | Read mapping recall TP/(TP+FN) |
| 19 | Read mapping F-score 2pr/(p+r) |
| 20 | % Well mapped but partial TP2/(TP1+TP2) |
| (21) | ALIGNMENTS |
| 22 | Misalignments some partial, FP=FP1+FP2 |
| 23 | Overlapping the truth, but extending out, FP1 |
| 24 | Mis-mappings not overlapping the truth, FP2 |
| 25 | Exact alignments identical to benchmark, TP1 |
| 26 | Exact but partial alignments, TP2 |
| 27 | Correct alignments, some partial, TP=TP1+TP2 |
| 28 | Unaligned, FN |
| 29 | Alignment mapping precision TP/(TP+FP) |
| 30 | Alignment mapping recall TP/(TP+FN) |
| 31 | Alignment mapping F-score 2pr/(p+r) |
| 32 | % Well mapped but partial TP2/(TP1+TP2) |

| **Species** | **Run** | **Methods** |
| --- | --- | --- |
| HG19 | T1R1 | 10_MagicBLAST,21_HISAT2,30_STAR,31_STARlong,32_STAR.2.6c,40_TopHat2 |
| HG19 | T1R1 truncated to 50 | 10_MagicBLAST,21_HISAT2,30_STAR,31_STARlong,32_STAR.2.6c,40_TopHat2 |
| HG19 | T1R2 | 10_MagicBLAST,21_HISAT2,30_STAR,31_STARlong,32_STAR.2.6c,40_TopHat2 |
| HG19 | T1R3 | 10_MagicBLAST,21_HISAT2,30_STAR,31_STARlong,32_STAR.2.6c,40_TopHat2 |
| HG19 | T2R1 | 10_MagicBLAST,21_HISAT2,30_STAR,31_STARlong,32_STAR.2.6c,40_TopHat2 |
| HG19 | T2R2 | 10_MagicBLAST,21_HISAT2,30_STAR,31_STARlong,32_STAR.2.6c,40_TopHat2 |
| HG19 | T2R3 | 10_MagicBLAST,21_HISAT2,30_STAR,31_STARlong,32_STAR.2.6c,40_TopHat2 |
| HG19 | T3R1 | 10_MagicBLAST,21_HISAT2,30_STAR,31_STARlong,32_STAR.2.6c,40_TopHat2 |
| HG19 | T3R2 | 10_MagicBLAST,21_HISAT2,30_STAR,31_STARlong,32_STAR.2.6c,40_TopHat2 |
| HG19 | T3R3 | 10_MagicBLAST,21_HISAT2,30_STAR,31_STARlong,32_STAR.2.6c,40_TopHat2 |
| Malaria | T1R1 | 10_MagicBLAST,21_HISAT2,30_STAR,31_STARlong,32_STAR.2.6c,40_TopHat2 |
| Malaria | T1R1 1/100 subsample | 10_MagicBLAST,21_HISAT2,30_STAR,31_STARlong,32_STAR.2.6c,40_TopHat2 |
| Malaria | T1R2 | 10_MagicBLAST,21_HISAT2,30_STAR,31_STARlong,32_STAR.2.6c,40_TopHat2 |
| Malaria | T1R3 | 10_MagicBLAST,21_HISAT2,30_STAR,31_STARlong,32_STAR.2.6c,40_TopHat2 |
| Malaria | T2R1 | 10_MagicBLAST,21_HISAT2,30_STAR,31_STARlong,32_STAR.2.6c,40_TopHat2 |
| Malaria | T2R2 | 10_MagicBLAST,21_HISAT2,30_STAR,31_STARlong,32_STAR.2.6c,40_TopHat2 |
| Malaria | T2R3 | 10_MagicBLAST,21_HISAT2,30_STAR,31_STARlong,32_STAR.2.6c,40_TopHat2 |
| Malaria | T3R1 | 10_MagicBLAST,21_HISAT2,30_STAR,31_STARlong,32_STAR.2.6c,40_TopHat2 |
| Malaria | T3R2 | 10_MagicBLAST,21_HISAT2,30_STAR,31_STARlong,32_STAR.2.6c,40_TopHat2 |
| Malaria | T3R3 | 10_MagicBLAST,21_HISAT2,30_STAR,31_STARlong,32_STAR.2.6c,40_TopHat2 |
| Human | iRefSeq | 10_MagicBLAST,20_HISAT2_relaxed,21_HISAT2,31_STARlong,32_STAR.2.6c,40_TopHat2 |

Table S6.2: Content of the lines in Excel Table S6. Table S6 includes 19 benchmark datasets in the order above, each of the Baruzzo benchmark has been tested with 6 methods: Magic-BLAST, HISAT2, STAR 1-pass, STAR long, STAR 2-pass and TopHat. iRefSeq has been tested also with HISAT relaxed, and the results for STAR 1-pass and TopHat have been omitted from the table.

## 7- Alignment statistics and the results of AliQC

Composite Excel Table S7 reports selected results from AliQC. Table S7.1 (below) shows the content of the output of the AliQC program, which gives a detailed panorama of the properties of the reads and their alignments. Only some of the results were discussed in the main article, so in excel Table S7, some of the columns are not reported. The first group of columns (listed in the left part of the table above) shows the alignment statistics: aligned reads, aligned length, multiplicity, strand, compatible pairs etc. The titles of each column should be self-explanatory. A second group of columns (center above) reports on the number of mismatches by type, by kb aligned, and the histogram of mismatches per aligned read. The third part of the AliQC report (to the right in Table S7.1, skipped in Table S7) gives the mismatches per position along the reads, i.e. per sequencing cycle.

Composite Excel table S7 reports on the same 16 datasets, treated with the same list of methods, altogether 92 experiments.

As shown in section S2, the percent reads-aligned is easy to measure, but cannot be used as a quality measure because different programs have different views of what an alignment is. In the Baruzzo 100+100 benchmark, a program such as TopHat reports only full-length alignments while Magic-BLAST is confident to report alignments down to 20 bases. In human RNA-seq, because introns leads to discontinuous alignments and a short segment is more difficult to map, such constraints may artificially disadvantage TopHat or Hisat and advantage Magic-BLAST or STAR. A more relevant measure is the total number of bases aligned or aligned uniquely. Another factor that may influence the measures is the maximal number of mismatches per kb accepted by each program. Programs like TopHat and HISAT, which only accept few mismatches per read, necessarily map a lower fraction of bases in samples with higher rate of mismatches. All these arguments explain why it is difficult to gauge all aligners on a single score. The multitude of quality controls computed by AliQC gives a more complete story.

Another way to “normalize” the measures is to impose a minimal alignment length. We present, in excel table S7, 3 sets of tables: all alignments (blue), then only those at least 50 (yellow) or at least 80 bases long (pink). All shorter alignments are skipped. We may all agree that, in human, an exact alignment of 50 bases with 20 base entropy is mostly unique.

Magic-BLAST aligns more reads and more bases than all other programs for all experimental datasets, and for all artificial datasets except in the simulated Human T1 and T2 where star-2pass is half a percent better.

Magic-BLAST also has fewer ambiguous mappings in all datasets except Roche, where Hisat2 is better by 1.5%.

The panorama is almost unchanged when we limit to at least 50 aligned bases.

Magic-BLAST keeps some very short alignments (as shown in section S2) and might ‘win’ at alignment efficacy because of this. But removing the alignments shorter than 50 bases in all programs, or even those shorter than 80 bases (to compare fairly to HISAT or TopHat, which require long alignments) is only marginally impacting the programs order and Magic-BLAST remains in the very top.

The rate of errors show that TopHat and HISAT2 have low tolerance to errors and this has consequences on alignment efficacy. The best representation we could think of for the quality of the aligner is presented in figure S7.1. For each dataset and each program. This figure shows the number of match bases versus the number of mismatches. The best aligner will in all case be the one with the largest number of matched baes, i.e. the most to the right, and the number of mismatches it found will be closes to the real number of mismatches in the dataset, which can be computed in mismatches per kilobase aligned. The aligners are color-coded, and the winning program is labelled by this rate. The dark-blue spot corresponds to the truth, when applicable, and by construction it always wins. In the 8 real datasets Magic-BLAST always fares bes except in Illumina 100+100 where STAR accepts more errors and aligned marginally more bases. Generally speaking, STAR tends to have more mismatches than all other program, whereas TopHat is always in the top left corner, because it aligns the fewer bases but so stringently that it has very few mismatches. In iRefSeq, STAR aligns 50% less bases than Magic-BLAST, again with many more mismatches. In the long reads, Hisat-relaxed is more successful than HiSat2.

In the long Illumina runs 250+250 and 300+300 Magic-BLAST wins by a very large margin. It matches 30% more bases than STAR, yet with less mismatches, and Hisat a distant third.

On the 3 PacBio, Magic-BLAST again wins. The striking lower number of errors on runs 52 and 67 is due to the fact that ducon did not submit his experimental data to SRA. He edited the reads using a separate Illumina run, fixing 10 mismatches per kilo-bases. This was not spotted at submission time and the data were accepted by error by NCBI.

In T3, Magic-BLAST is a little better than STAR, where as Tophat and Hisat fail lamentably; no program accepts the true rate of error. In T2, STAR has a small lead over Magic-BLAST, and both are close to the error rate of the truth. In T1, Star is best, followed by Hisat than Magic-Blast in human, in malaria by magic-BLAST then Hisat.

The discrepancy between the real and the simulated datasets is most like due to the fact the simulated error models does not mimic the reality, in particular, the Baruzzo data have frequent relatively long stretches of inserted od deleted bases.

| **Alignment statistics: aligned reads, aligned bases, alignment length, multiplicity, strand, compatible pairs** | **Mismatches statistics: number of mismatches by type, by kb aligned, histogram of mismatches per aligned read** | **Mismatches per position along the reads, i.e. per sequencing cycle (AliQC result omitted from table S7)** |
| --- | --- | --- |
| Read | Read | Run |
| Run | Run | Method |
| Method | Method | Aligned bases |
| % aligned reads | Reads in run or in file | Aligned bases in read 1 |
| % bases aligned | % reads aligned with no mismatch | Aligned bases in read 2 |
| % mapped pairs | % reads with 0 or 1 mismatch | Rate of Mismatch per Mb per sequencing cycle in read 1 |
| % compatible pairs* | Aligned bases | Cycle 1 |
| % fragments mapped on plus strand** | mismatches per kb aligned | **Cycle 2 to 205** |
| % aligned reads with unique alignments | substitutions per kb | Cycle 205 |
| % aligned reads with multiple alignments | transitions per kb | Rate of Mismatch per Mb per sequencing cycle in read 2 |
| Reads in run or in file | transversions per kb | Cycle 1 |
| Reads unaligned | insertions per kb | **Cycle 2 to 205 (1 column per base position)** |
| Reads aligned | deletions per kb | Cycle 205 |
| Reads with several alignments*** | Reads with 0 mismatch |  |
| Reads with unique alignments | **1 mismatch to 100 mismatches (100 columns)** |  |
| **Reads with 2 alignments, 3… 80 alignments** | Mismatches |  |
| … Reads with 80 alignments | Substitutions |  |
| Fragments aligned on plus strand** | Transitions |  |
| Fragments aligned on minus strand** | Transversions |  |
| Pairs with both ends aligned | Insertions |  |
| Compatible pairs | Deletions |  |
| Raw bases in unaligned reads present in the file | A>G |  |
| Raw bases in aligned reads | T>C |  |
| Aligned bases | G>A |  |
| (histogram of length aligned, not shown in S7, replaced by Excel Table S2) | C>T |  |
|  | A>T |  |
|  | T>A |  |
|  | G>C |  |
|  | C>G |  |
|  | A>C |  |
|  | T>G |  |
|  | G>T |  |
|  | C>A |  |
|  | Ins A |  |
|  | Ins T |  |
|  | Ins G |  |
|  | Ins C |  |
|  | Del A |  |
|  | Del T |  |
|  | Del G |  |
|  | Del C |  |

Table S7.1 Content of the columns in the output of the AliQC program.

### 7.2 Matches and mismatches, profile of insertions, deletions, transitions and transversions

### 7.3 Compatible pairs, reads with unique or multiple alignments


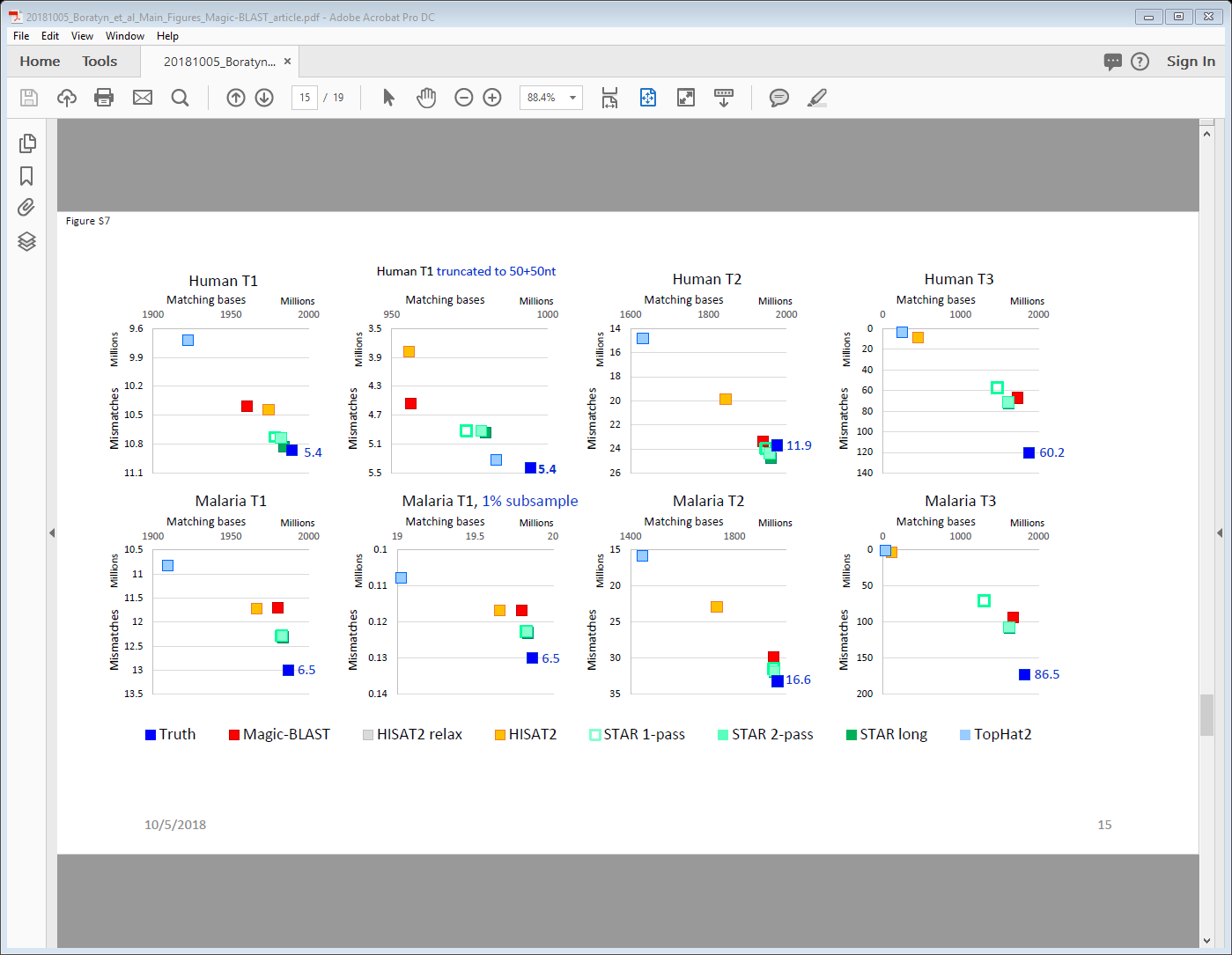
Figure S7.1 Matches and mismatches in the Baruzzo benchmark
